# Impact of Transcatheter Edge-to-Edge Repair on Tricuspid Annular Remodeling in Patients with Tricuspid Regurgitation

**DOI:** 10.3390/jcm14155606

**Published:** 2025-08-07

**Authors:** Maddalena Widmann, Roberto Nerla, Fausto Castriota, Andrea Fisicaro, Valeria Maria De Luca, Gabriele Pesarini, Flavio Luciano Ribichini, Angelo Squeri

**Affiliations:** 1 Division of Cardiology, Department of Medicine, University of Verona, 37134 Verona, Italy; 2 Cardiology Unit, Maria Cecilia Hospital GVM Care and Research, 48033 Cotignola, Italy

**Keywords:** tricuspid regurgitation, transoesophageal echocardiography, edge-to-edge repair, tricuspid annulus

## Abstract

**Background:** In recent years, multiple transcatheter devices have been developed for tricuspid valve intervention. The aim of this study was to evaluate acute tricuspid annular remodeling following percutaneous leaflet repair using a leaflet approximation device for the reduction of tricuspid regurgitation (TR). **Methods:** This retrospective cohort study included 26 consecutive patients treated at two centers. Tricuspid annular geometry was assessed using three-dimensional transesophageal echocardiography during the procedure. **Results:** The mean age of the cohort was 79.3 years, and 88.5% were female. All patients had severe or greater TR pre-procedure, mostly due to annular dilation. The procedure was successful in all cases, with at least a one-grade reduction in TR observed prior to hospital discharge. Significant reductions were observed in the mean septal-lateral diameter (4.09 ± 0.44 cm vs. 3.54 ± 0.53 cm, *p* < 0.0001), mean major diameter (4.65 ± 0.63 cm vs. 4.28 ± 0.65 cm, *p* = 0.0002), planimetric area (14.00 ± 2.91 cm^2^ vs. 11.25 ± 2.91 cm^2^, *p* < 0.0001), and perimeter (13.62 ± 1.43 cm vs. 12.42 ± 1.62 cm, *p* < 0.0001) of the tricuspid annulus. **Conclusions:** In this small real-world cohort, transcatheter edge-to-edge repair was found to be both effective and safe. The use of a leaflet approximation device not only reduced TR severity but also led to significant reductions in annular dimensions. To our knowledge, this study provides additional evidence of acute tricuspid annulus remodeling following edge-to-edge repair, which may have significant therapeutic implications.

## 1. Introduction

Clinically relevant tricuspid regurgitation (TR) is a prevalent valvular disease affecting approximately 3.0 million individuals in Europe and 1.6 million in the United States [1]. TR is classified according to the underlying mechanism into primary, secondary and isolated. Severe TR has significant prognostic implications, including a negative impact on survival and worsening heart failure [1,2]. A retrospective analysis of a large cohort of patients shows one-year mortality rates of 29.5% for moderate TR and 45.6% for severe TR, independently of potential confounders. Also, the rate of heart failure hospitalization is positively correlated with TR severity [3].

Transthoracic echocardiography plays a crucial role in diagnosing TR, enabling the quantification of regurgitation severity, differentiation between primary and secondary TR, assessment of associated left-sided diseases, and evaluation of right ventricular (RV) size and function [3,4]. The grading of TR, traditionally definite as mild, moderate and severe, requires a comprehensive, multi-parametric approach and the traditional classification of TR into mild, moderate, and severe categories has been expanded with the addition of two new grades: “massive” and “torrential”, which are now commonly used in clinical studies to quantify the impact of transcatheter interventions [5]. Some studies have shown that a reduction in TR by at least one grade is clinically significant and correlates with improvements in quality of life [6]. Recently, a study from the Triluminate Trial also showed a significant reduction in heart failure hospitalization for patients treated with T-TEER compared to medical therapy alone [7].

The tricuspid valve (TV) has a complex anatomical structure, and the interdependence between TV and RV explains the physiopathological mechanisms of most forms of TR. The tricuspid annulus (TA) is a nonplanar structure, with a more ventricular posteroseptal portion and a more atrial anteroseptal portion, near the RV outflow tract and the aortic valve. A study demonstrated that in patients with functional TR, TA has a more circular shape resulting from dilation in the septal to lateral and posteroseptal to anterolateral directions. Moreover, the TA becomes more planar, the more severe the TR [8].

Nowadays, right atrial and ventricular geometry in patients with TR is accurately evaluated to better define the pathophysiology and clinical implications of the valvular disease.

Despite TR being substantially undertreated, a deeper understanding of its pathophysiology and prognostic implications has led to a shift from conservative to more interventional treatment strategies. In the last decades, multiple transcatheter devices for TV interventions have been developed and are now considered valuable treatment options in anatomically eligible patients at high surgical risk. Percutaneous devices involve similar techniques as surgical repair, and a recent analysis shows significant improvement in quality of life with fewer periprocedural complications [9]. Efficacy and clinical outcome are promising [10,11], but the effect on RV reverse remodelling is still debated.

Percutaneous techniques currently available for the treatment of tricuspid regurgitation are mainly derived from surgery and include the leaflet-approximation technique, which aims to reduce TR through leaflet grasping and the transcatheter annuloplasty. The most studied device for percutaneous annuloplasty is the Cardioband™ (Edwards Life-sciences, Irvine, CA, USA) system that addresses the pathophysiological mechanism of annular dilatation and consists of the implantation of an adjustable band on the TA using several screws as anchors.

The TriClip (Abbott Vascular, Santa Clara, CA, USA) and the PASCAL (Edwards Lifescience, Irvine, CA, USA) are transcatheter leaflet repair systems that act by approximating tricuspid leaflets, and in a propensity-matched analysis, their outcomes are comparable [12]. The technique consists of anterior to septal or posterior to septal leaflet approximations or a combination of both. In exceptional cases, a “clover technique” may be used [13]. Real-world analyses demonstrate a high procedural success rate with a reduction in TR of at least one grade in most patients [14,15]. RV remodelling after percutaneous TV interventions has to date few evidences, but the available data are promising [9,16]. Analysis of the hemodynamic consequences of transcatheter tricuspid edge-to-edge repair in a patient with isolated TR shows that reduction of regurgitant volume leads to a decrease of RV volume overload with reduction in RV diastolic dimensions and total RV stroke volume [17]. On the other hand, this causes an augmentation of pulmonary forward flow with subsequent enhanced left ventricular filling and improvements in cardiac index. Also, a reduction in external work of the RV is observed, with improvement of RV performance and reduction of oxygen consumption [18].

The aim of the study is to evaluate the acute TA remodelling after percutaneous leaflet repair. Authors wanted to test if the leaflet approximation technique can also lead to a reverse TA remodelling as a potential mechanism for TR reduction.

## 2. Methods

### 2.1. Study Population

This retrospective cohort study included 31 consecutive symptomatic patients with TR ≥ severe treated with TriClip System at the Maria Cecilia Hospital in Cotignola and the University Hospital of Verona between December 2017 and February 2023. Four patients were excluded for the absence of a complete 3D dataset of the tricuspid valve before and immediately after the procedure. One patient was excluded because the 3d dataset was of inadequate quality for annular dimension analysis. In the end, 26 patients were included in the present study. Two different interventional cardiologists performed the procedures as the first operator. Demographic data, cardiovascular risk factors, and blood samples were collected at admission (Table 1).

### 2.2. Echocardiographic Evaluation

Pre- and post-procedural echocardiographic parameters, using transthoracic and transesophageal echocardiography, were obtained (Table 2). All echocardiograms were performed on an IE33 or EpiqCVX (Philips Medical System, Andover, MA, USA) according to current guidelines by the European Association of Cardiovascular Imaging and American Society of Echocardiography.

TR severity was quantified using a combination of qualitative, semiquantitative, and quantitative methods, employing a five-grade scale. The aetiology of TR was categorized as primary, functional atrial, functional ventricular, or lead-associated.

For each patient, a Zoom-3D echocardiographic data set was acquired, with depth and sector adjustments to capture the TV apparatus within the data set as much as possible while maximizing spatial resolution. Single-beat acquisition was used in all patients to prevent stitching artifact due to atrial fibrillation, respiratory, or probe movements. No minimum frame rate was required.

All the TEE 3D examinations used for evaluation of TA were performed by the same trained echocardiographer before and after Triclip implant. They were conducted during the procedure in fasting patients after the induction of general anaesthesia. The reconstructions were analysed using the QLab v.10 (Philips Medical Systems, Andover, MA, USA) software package from stored images by an experienced operator blinded to procedural details. Procedural data were also collected (Table 3).

Intraprocedural success was defined according to the Tricuspid Valve Academic Research Consortium (TVARC) definitions [19]. Specifically, acute procedural success (APS) was defined as successful implantation of the device resulting in TR less than or equal to moderate (≤2+).

The study was conducted in accordance with the ethical principles of the Declaration of Helsinki, and all the patients included entered in the prospective clinical registry and provided their written consent for the anonymous collection of the data.

### 2.3. Tricuspid Annulus Analysis

**Figure 1:**
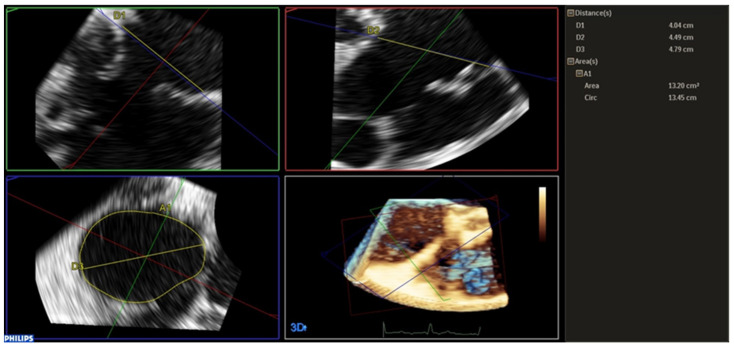
Tricuspid annular measurement, Tricuspid annular measurements using multi-planar reconstruction (MPR) from 3D transesophageal echocardiography. The septal-lateral and antero-posterior diameters, as well as annular area and perimeter, were assessed at end-diastole using orthogonal planes and planimetric tracing.

The QLab v.10 software was used to analyse TA from intraprocedural 3D examinations. Manipulation of the 3D data sets by multiplanar reconstruction (MPR) was performed. The 3D data sets were analysed offline using a multiplanar reconstruction (MPR) analysis. The full-volume acquisitions were offline manually manipulated to identify 2D cut planes corresponding to the transverse coronal and sagittal planes at the level of the tricuspid annulus to accurately identify the maximal septal-lateral (SL) and antero-posterior (AP) diameters and to calculate the perimeter and area of the TA. Also, the major diameter of the planimetric TA was measured. Analysis was conducted at the end-diastole. The SL and AP dimensions were measured respectively in the coronal and the sagittal plane, using some landmarks, such as the aortic root, for correct alignment. The TA was planimetered in the transverse plane to obtain perimeter, area, and major diameter (Figure 1). For all patients, the same analysis is performed from data sets collected before and after TV repair, using the same method. The eccentricity index was obtained by dividing the SL diameter by the AP diameter. Measurements were obtained at the end-diastole before and after the intervention.

### 2.4. Statistical Analysis

For descriptive analyses, categorical variables are presented as numbers and percentages, and continuous variables are presented as means ± standard deviation. The parameters of AP, SL, major diameter, as well as area and perimeter of the TA were compared before and after TriClip implantation using a *t*-test for paired data. Eccentricity index was also compared using the same statistical method. All data were processed using the Statistical Package for Social Sciences, version 24 (SPSS, Chicago, IL, USA).

## 3. Results

### 3.1. Clinical and Echocardiographic Characteristics

The study population includes 26 subjects with at least severe tricuspid regurgitation. The mean age of the study cohort was 79.3 years, and 88.5% were female. Particularly, 46.2% of patients had severe TR, 11.5% massive, and 42.3% torrential TR. The mechanism of TR was atrial functional in 21 (80.8%) cases, ventricular in 2 (7.7%), primary in 1 (3.8%) case, and lead-associated in 2 (7.7%) due to leaflet impingement. The majority (69.2%) of patients had central and antero-septal dominant regurgitant jet (18). Six patients (23.1%) had a wide regurgitant jet from the postero-septal to the antero-septal commissure. Only two patients had only postero-septal dominant regurgitant jet (7.7%). Severe functional limitation (New York Heart Association ≥ III) was seen in 69.2% of patients. A total of 8 patients had prior valve intervention, and atrial fibrillation (AF) was identified preoperatively in 92.3% (*n* = 24) of patients. A total of 4 patients had prior PMK/ICD implantation, and 8 had coronary artery disease.

At admission, a significant portion (42.3% *n* = 11) had peripheral oedema, and all patients were on diuretic therapy. A total of 8 (30.8%) had one or more previous hospitalizations for heart failure, and the mean value of NT-proBNP was 3234 pg/mL. At pre-operative transesophageal and transthoracic echocardiographic examination, RV systolic dysfunction was identified in 3 patients (11.5%), with a mean fractional area change of 41.43 ± 5.69% and a mean tricuspid plane systolic excursion of 18.08 ± 2.07 mm. The mean pulmonary systolic pressure was 49.65 ± 16.59 mmHg. Right heart catheterization was performed in all cases to exclude severe or pre-capillary pulmonary hypertension. All patients had preserved left ventricular ejection fraction, and 3 (11.5%) of them had moderate-severe mitral regurgitation (Table 2).

### 3.2. Operative Outcomes

Edge-to-edge reparation was performed in all patients (*n* = 26) with at least a 1-grade reduction of TR at the end of the procedure. The procedural success was obtained in 24 cases (92.31%). Most patients (84.6% *n* = 22) implanted two clips. Only 2 (7.7%) patients were implanted with 1 or 3 clips. The device was placed between the anterior and the septal leaflets in 76.9% of patients and between the posterior and the septal leaflets in 15.4% of cases. Only 7.7% of the subjects received combined antero–septal and postero–septal clip approximation.

New permanent pacemaker implantation was required in 1 patient for a second-degree atrioventricular block, and another patient had a vascular complication of the operative access. The median length of a hospital stay was 5 days (Table 3).

### 3.3. Echocardiographic Outcomes

**Figure 2:**
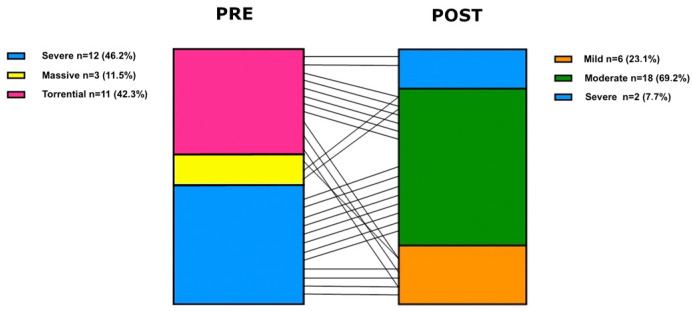
Tricuspid regurgitation quantification pre- and post-intervention.The figure shows the distribution of TR severity before and after the intervention, with the number of patients classified as having severe, massive, or torrential TR pre-intervention, and the number of patients with mild or greater TR post-intervention.

Before hospital dismissal, 23.1% (*n* = 6) of patients had mild TR (Table 4). TR grade was reduced to at least moderate in all but 2 (7.7%) patients. There was no development of new regurgitant jets except for two patients with an unsuccessful procedure. In one case, severe distortion of tricuspid valve anatomy and in the second one, a small iatrogenic flail were the causative factors. In all the other cases, we had no development of new regurgitant jets but a reduction of pre-existing jets. All patients (100% *n* = 26) had at least a 1-grade reduction of TR (Figure 2). Mean tricuspid plane systolic excursion was 17.29 ± 3.58, and mean left ventricle ejection fraction was 59.96 ± 8.83%.

### 3.4. Acute Tricuspid Annulus Remodelling

**Figure 3:**
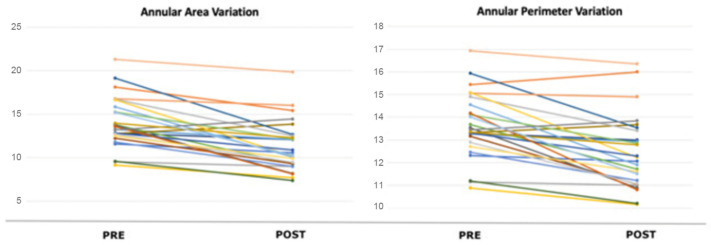
Variation in tricuspid annular area and perimeter pre- and post-procedure in every single patient. The graph displays the change in both area and perimeter of the tricuspid annulus, comparing measurements before and after the intervention for each patient in the study cohort.

Analysis of the TA dimension before and after TriClip implantation (Table 5) showed a significant reduction of the mean SL diameter (*p* ≤ 0.0001) and of the mean major diameter (*p* = 0.0002), whereas the reduction of the AP diameter did not reach statistical significance (*p* = 0.0626). Comparing the planimetric area and perimeter of the annulus (Figure 3) before and after the procedure, a significant reduction was observed (*p* ≤ 0.0001, *p* ≤ 0.0001). Finally, the mean eccentricity index was 0.98 ± 0.17 before TriClip implantation and 0.90 ± 0.17 after (*p* = 0.0286).

## 4. Discussion

Both the TRILUMINATE Pivotal (Trial to Evaluate Cardiovascular Outcomes in Patients Treated with the Tricuspid Valve Repair System) and the CLASP TR (Edwards PASCAL Transcatheter Valve Repair System in Tricuspid Regurgitation) trials demonstrated not only a significant reduction in TR grade after T-TEER, but also a significant decrease in SL diameter of the TV annulus within the first 30 days post-procedure, with sustained remodelling at follow-up [20,21,22]. A study by Russo et al. [23] also demonstrated significant acute changes in the TV annulus geometry following T-TEER in the TriValve population. One major limitation of those studies is that the SL diameter of the TV annulus was investigated only by 2D TTE, which is known to have major limitations in the evaluation of TV annulus maximal diameter. Recently, a monocentric study from Cammalleri et al. [24] showed a reshaping of the tricuspid annulus immediately after T-TEER evaluated by 3D echo.

In this retrospective, dual-center cohort study, TA dimensions were analysed before and early after edge-to-edge tricuspid leaflet repair using TriClip in a real-world population. The study population is composed of older people, mostly women, with atrial functional TR. Only a few patients have associated left-sided valvular or ischemic disease, and none have left ventricular systolic dysfunction. However, almost all patients have anamnestic AF, and so the predominant mechanism of TR in the population is annular dilation and right atrial remodelling secondary to AF. The study population is comparable with those of the pivotal studies of the TriClip and PASCAL systems, and because of the prohibitive surgical risk due to age, comorbidities, and isolated tricuspid valve disease, these patients are good candidates for percutaneous therapies [25,26].

The study demonstrates a procedural success in most of the patients, with correct implantation of one or more devices. No life-threatening complications are observed, confirming a good safety profile of the system. A five-class grading scheme is used in the pre-operative echocardiographic transthoracic and transesophageal evaluation to assess the severity of TR. Before dismissal, all patients showed a reduction of TR of at least 1 grade, and only four of them had severe residual TR. In addition, no patient developed acute right ventricular dysfunction.

TR caused by annular dilation has been surgically treated for decades by ring annuloplasty. Edge-to-edge percutaneous systems are derived from surgery for mitral regurgitation treatment, and then applied also on the right side [22]. TriClip and the other available edge-to-edge reparation systems reduce valve regurgitation by stitching together the tricuspid leaflets and limiting their motion. The reduction of volume overload generates a positive reverse remodelling of the RV. This observational study sought to evaluate the acute remodelling of the TA due to direct traction on the leaflets after TriClip implant. The retrospective analysis of 3D echocardiographic intraprocedural evaluations demonstrated that edge-to-edge tricuspid reparation using TriClip results in a positive acute remodelling of the TA, with significant reduction of the SL and the major diameter. This finding can be clearly understood by the fact that only the septal and anterior leaflets and the septal and posterior leaflets can be stitched together. Also, planimetric area and perimeter decreased significantly, corroborating the echocardiographic outcomes. The analysis of the AP diameter highlights a reduction that does not reach statistical significance, and this result could be associated with the evidence of a decrease in the eccentricity index. In fact, a study demonstrated that TA dilates mostly at the lateral region, because the septal region is more rigid and fibrous. For this reason, the coaptation gap is often located between the anterior and septal leaflets, and most of the clips were placed in the antero-septal coaptation line (Table 3). It could have twisted the TA in some patients, giving it a more oval shape and decreasing its eccentricity.

## 5. Limitations

Several limitations should be acknowledged. First, this study was performed retrospectively, based on a small sample size cohort without any control subjects. Therefore, these results need to be confirmed in a larger, prospective study. Furthermore, the quality of intraprocedural imaging can be suboptimal because of shadowing by intracardiac implants or by the TriClip device itself, and this aspect could affect the analysis with the reconstruction software. About this, all measurements were made by a single operator, and intra- and inter-observer variability were not assessed. In addition, no prognostic implications were provided.

## 6. Conclusions

In this small real-world population, edge-to-edge repair using TriClip was found to be effective and safe. Our data support the presence of acute remodelling of the tricuspid annulus due to direct traction of the implanted clips on the leaflet, with a significant reduction of SL diameter.

## Figures and Tables

**Table 1:** Demographic variables, cardiovascular risk factors and blood samples at admission.

Parameters	All Patients (*n* = 26)
Age (years)	79.3 (64–88)
Female Sex	23 (88.5%)
Body mass index (kg/m^2^)	25.2 (16.7–32.9)
Arterial Hypertension	18 (69.2%)
Diabetes	2 (7.7%)
Dyslipidemia	14 (53.8%)
Smoke	1 (3.8%)
NYHA Class	
I	0 (0%)
II	8 (30.8%)
III	17 (65.4%)
IV	1 (3.8%)
Coronary Artery Disease	8 (30.8%)
PCI	5 (19.2%)
CABG	1 (3.8%)
Valvular Intervention	8 (30.8%)
Aortic Valve Replacement	2
Mitral Valve Replacement	4
Mitral Valve Repair	2
Tricuspid Valve Repair	1
Mitraclip	1
Atrial Fibrillation	24 (92.3%)
PMK/ICD	4 (15.4%)
COPD	3 (11.5%)
HF Hospitalization	8 (30.8%)
Peripheral Edema	11 (42.3%)
Diuretics	26 (100.0%)
Hemoglobin (g/dL)	12.51 ± 1.49
CKD (eGFR < 60 mL/min)	15 (57.7%)
Creatinine (mg/dL)	1.08 ± 0.40
NTproBNP (pg/mL)	3234 ± 2948

NYHA: New York Heart Association, PCI: percutaneous coronary intervention, CABG: coronary artery bypass graft, PMK: Pacemaker, ICD: Intracardiac Defibrillator, COPD: Chronic Obstructive Pulmonary Disease, HF: Heart Failure, CKD: Chronic Kidney Disease, eGFR: Glomerular Filtration Rate.

**Table 2:** Pre-operative echocardiographic examination.

Parameters	All Patients (*n* = 26)
Sinus rhythm	3 (11.5%)
Atrial Fibrillation/Atrial Flutter	22 (84.7%)
Pacemaker rhythm	1 (3.8%)
EDA (cmq)	21.15 ± 6.06
FAC (%)	41.43 ± 5.69
TAPSE (mm)	18.08 ± 2.07
PAPS (mmHg)	49.65 ± 16.59
EF (%)	58.67 ± 7.14
Tricuspid Regurgitation	
Mild	0 (0%)
Moderate	0 (0%)
Severe	12 (46.2%)
Massive	3 (11.5%)
Torrential	11 (42.3%)
Primary Mechanism	
Atrial Functional	21 (80.8%)
Ventricular Functional	2 (7.7%)
Primary	1 (3.8%)
Lead associated	2 (7.7%)
Mitral Regurgitation	
Mild	11 (42.3%)
Moderate	7 (26.9%)
Moderate-Severe	3 (11.5%)
Aortic Regurgitation	
Mild	13 (50.0%)
Moderate	4 (15.4%)
Severe	0 (0%)

**Table 3:** Operative Outcomes.

Parameters	All Patients (*n* = 26)
Clip (*n*)	
1	2 (7.7%)
2	22 (84.6%)
3	2 (7.69%)
A-S	76.9%
P-S	15.4%
A-P-S	7.7%
A-P	0%
Success	26 (100%)
Complications	2 (7.7%)
Death	0
Hospitalization Length (days)	5

A = anterior leaflet, S = septal leaflet, P = posterior leaflet.

**Table 4:** Echocardiographic outcomes.

Parameters	All Patients (*n* = 26)
Tricuspid regurgitation	
Mild	6 (23.1%)
Moderate	18 (69.2%)
Severe	2 (7.7%)
TAPSE (mm)	17.29 ± 3.58
PAPS (mmHg)	44.04 ± 12.85
EF (%)	59.96 ± 8.83

**Table 5:** Acute tricuspid annulus remodelling.

Parameters	Before	After	*p* Value
SL diameter (cm)	4.09 ± 0.44	3.54 ± 0.53	<0.0001
AP diameter (cm)	4.29 ± 0.79	4.01 ± 0.62	0.0626
Major diameter (cm)	4.65 ± 0.63	4.28 ± 0.65	0.0002
Area (cmq)	14.00 ± 2.91	11.25 ± 2.91	<0.0001
Perimeter (cm)	13.62 ± 1.43	12.42 ± 1.62	<0.0001
Eccentricity Index	0.98 ± 0.17	0.90 ± 0.17	0.0286

## Data Availability

The data presented in this study are available upon request from the corresponding author. The data are not publicly available due to the Data Protection Directive 95/46/EC.

## Abbreviations

The following abbreviations are used in this manuscript:

TR	Tricuspid Regurgitation
TA	Tricuspid Annulus
RV	Right Ventricle
TV	Tricuspid Valve
3D	Three Dimensional
CMR	Cardiac Magnetic Resonance
TEE	Transesophageal Echocardiogram
AF	Atrial Fibrillation
